# High Tolerance to Salinity and Herbivory Stresses May Explain the Expansion of *Ipomoea Cairica* to Salt Marshes

**DOI:** 10.1371/journal.pone.0048829

**Published:** 2012-11-15

**Authors:** Gang Liu, Qiao-Qiao Huang, Zhen-Guang Lin, Fang-Fang Huang, Hui-Xuan Liao, Shao-Lin Peng

**Affiliations:** 1 State Key Laboratory of Biocontrol and Guangdong Key Laboratory of Plant Resources, School of Life Sciences, Sun Yat-Sen University, Guangzhou, China; 2 Environment and Plant Protection Institute, Chinese Academy of Tropical Agricultural Sciences, Danzhou, China; University of Konstanz, Germany

## Abstract

**Background:**

Invasive plants are often confronted with heterogeneous environments and various stress factors during their secondary phase of invasion into more stressful habitats. A high tolerance to stress factors may allow exotics to successfully invade stressful environments. *Ipomoea cairica*, a vigorous invader in South China, has recently been expanding into salt marshes.

**Methodology/Principal Findings:**

To examine why this liana species is able to invade a stressful saline environment, we utilized *I. cairica* and 3 non-invasive species for a greenhouse experiment. The plants were subjected to three levels of salinity (i.e., watered with 0, 4 and 8 g L^−1^ NaCl solutions) and simulated herbivory (0, 25 and 50% of the leaf area excised) treatments. The relative growth rate (RGR) of *I. cairica* was significantly higher than the RGR of non-invasive species under both stress treatments. The growth performance of *I. cairica* was not significantly affected by either stress factor, while that of the non-invasive species was significantly inhibited. The leaf condensed tannin content was generally lower in *I. cairica* than in the non-invasive *I. triloba* and *Paederia foetida*. *Ipomoea cairica* exhibited a relatively low resistance to herbivory, however, its tolerance to stress factors was significantly higher than either of the non-invasive species.

**Conclusions/Significance:**

This is the first study examining the expansion of *I. cairica* to salt marshes in its introduced range. Our results suggest that the high tolerance of *I. cairica* to key stress factors (e.g., salinity and herbivory) contributes to its invasion into salt marshes. For *I. cairica*, a trade-off in resource reallocation may allow increased resources to be allocated to tolerance and growth. This may contribute to a secondary invasion into stressful habitats. Finally, we suggest that *I. cairica* could spread further and successfully occupy salt marshes, and countermeasures based on herbivory could be ineffective for controlling this invasion.

## Introduction

Biological invasions are recognized as a major driver of biodiversity decline and altered ecosystem services worldwide [1]. Invasive weeds can profoundly alter the structure and function of natural ecosystems [2], [3], [4]. Due to the ecological and economic impacts of invasive species, many scientists and managers are interested in predicting the environments that are most likely to be invaded by exotic species [5], [6]. Invasions are influenced by biotic factors (e.g., herbivory) [6], [7] and abiotic factors (e.g., salinity) [8], [9]. Local environments frequently change along gradients of certain factors (e.g., soil nutrient gradients and water gradients [10], [11]), even over short distances [12]. Many studies have examined the relative importance of abiotic factors on the distribution of organisms in heterogeneous environments [13], [14], [15]. The results of these studies indicate that the boundary of a population toward the more benign end of the gradient is usually the result of competition, whereas the population boundary toward the more stressful end is mainly controlled by abiotic factors [16].

Environments vary in their invasibility [17], and it may be more difficult for exotics to invade habitats with stressful factors. For example, it is likely that salt marshes [18] are rarely inhabited by non-natives due to their hyper-saline soil. Although stressful environments may prohibit most non-native invasions, some vigorous invaders can successfully invade stressful habitats [19], [20], [21]. It is possible that the primary invasions of these species occurred in benign habitats, and during their secondary invasions, the species expanded into stressful habitats [22].

Many hypotheses have been proposed for invasion mechanisms. The Enemy Release Hypothesis (ERH) [23] is one of the most important hypotheses, and it posits that one of the leading causes of the increased vigor of invaders in the introduced range is liberation from co-evolved natural enemies. Thus, invasive plants usually have lower levels of resistance than their conspecifics in their native ranges [24]. Resistance is costly [25], and decreased defense may help invaders to maintain fitness when they encounter competitors or stressful factors. The relationship between decreased resistance and increased competiveness of invaders has been demonstrated by many researchers [26], [27], [28]. However, an increasing number of recent studies have indicated that invasive plants are also subject to herbivory in introduced regions [24], [29], [30]. The notion that tolerance may also play an important role in facilitating successful invasions has become more widely accepted [31], [32], [33]. Indeed, the tolerance characteristics of an invasive species may help to ensure successful invasions [30].

Tolerance is the ability of a plant to maintain fitness when subjected to a stress factor [34] such as herbivory [35] or salinity [36], [37]. For many species, especially invasive species, there is usually more than one stress factor that must be dealt with simultaneously when they expand into new environments. Species under these conditions may exhibit reduced fitness, and in such cases, some species may not survive or spread. For example, species that are adapted to resource-poor environments grow inherently more slowly and are more sensitive to herbivory than species from richer habitats [38]. Leaf damage decreases fitness [39] and constrains the phenotypic plasticity of plant species exposed to drought [40].

*Ipomoea cairica* is a perennial herbaceous twining vine that originates from tropical Africa [41] and is listed as one of the most invasive species in South China [42]. The vine can invade bare land, wasteland and the edges of forests [21], although considerable herbivory pressure from a native insect has been observed. Recently, we found that this invasive species was expanding from its usual low-salinity habitats into the salt marshes between terrestrial and marine ecosystems. Salt marshes are characterized by high salinity, which usually inhibits the expansion of terrestrial plants. This high salt concentration not only decreases the osmotic potential of the soil creating water stress in plants [43], but it also causes severe ion toxicity because Na^+^ and Cl^−^ are not as readily sequestered into vacuoles as they are in halophytes [44], [45], [46]. In salt marshes, salinity can influence the growth of plants and may become a key restrictive factor for non-native plants, especially those originating from very different environments, which may be due to the large energy costs of excluding salt [47] and the associated osmotic adjustments [48]. Thus, salt marshes may not be easily invaded by non-native plants that grow in non-saline habitats. Given that certain non-native plants can establish their populations in this type of stressful habitat, they may have to allocate additional energy to balance the osmotic potential. Furthermore, if stressful herbivory occurred in addition to salt stress, the invasive species could exhibit decreased fitness in this stressful habitat and possibly fail to survive. Previous studies have commonly focused on the relationship between decreased resistance and increased competiveness of invaders in benign habitats, seldom focusing on the relationship between the resistance and tolerance of invaders in stressful habitats. Until now, there has been no research undertaken on the expansion of the invasive glycophyte *I. cairica* into a salinity-stressed habitat.

In a review, van Kleunen *et al.* [49] indicated that invasive species usually have higher values for performance-related traits than non-invasive species and that this could be used to predict plant invasion potential based on species traits. Exotics with higher values for these traits compared with native or non-native congeners may be more vigorous invaders. Thus, comparisons of the performance and traits of invasive and non-invasive species [49] across multiple environmental conditions [50] were applied to understand the means by which *I. cairica* expanded into salt marshes. We compared the performance of *I. cairica* with an exotic non-invasive species (*I. triloba*), a native species (*I. digitata*), and a native species that has invaded other continents (*Paederia foetida*). The invasive alien vs. native comparison is related to the invasion process, whereas the invasive alien vs. non-invasive alien comparison is related to the establishment of the alien species [49]. Although the trait differences between invasive and native species that have invaded other continents have not been shown to be significant [49], we assumed that the resistance characteristics may be different between them because the former might have been released from natural enemies for a long time, whereas the latter have not. We hypothesized that the successful expansion of the invasive species from its founding population to a novel stressful habitat benefits from its low level of resistance and high level of tolerance to primary stress factors. We addressed three main questions in this study: (1) Is the growth of the invasive species affected by simulated herbivory and salinity stress? (2) Does the invasive species have a higher tolerance to stress factors than the non-invasive species? (3) Does the invasive species exert lower resistance to herbivory than the non-invasive species, especially when herbivory pressure exists? Finally, we discuss the probability of successful invasion of salt marshes by *I. cairica*.

## Materials and Methods

### Materials

Four liana species were selected for this experiment, the invasive species *Ipomoea cairica*, the exotic non-invasive species *I. triloba* and two native species, *I. digitata* and *Paederia foetida*. The former three species are members of the Convolvulaceae family.

The earliest records of *I. cairica* in China date back to 1912 [51]. *Ipomoea cairica* usually invades farmlands, forest edges, roadsides and other habitats that lack salinity stress [21]. The small size and recent emergence of the salt marsh population suggest that it arose from the large older population in the surrounding forests. Furthermore, in its native range, *I. cairica* commonly grows in woodland and anthropic habitats [52] with no salinity stress.

*Ipomoea triloba*, which is native to tropical America, invades many Pacific islands. In these non-native ranges, it is usually found in grasslands, waste places, roadsides [53], and occasionally in habitats near sea level [54]. However, there is no evidence indicating that *I. triloba* grows in salinity-stressed habitats in its native range. *Ipomoea digitata* typically grows in forests and roadsides in South China, which is its native range. *Paederia foetida*, which is a member of the Rubiaceae, has significantly invaded the United States [55]. It is typically found in forest gaps and roadsides in native ranges and commonly co-occurs with *I. cairica* in South China.

The invasive species *I. cairica* reproduces primarily through vegetative propagation and spreads vegetatively [56], and it was difficult to collect enough seeds for this experiment. We therefore established invasive plants as vegetative cuttings, and the remaining three species were established from seeds. In November and December of 2010, we collected these seeds from six cities in the Guangdong province: Guangzhou, Zhuhai, Shenzhen, Foshan, Zhongshan and Zhaoqing. Most of these seeds were collected from non-saline habitats, and only a few of them were collected from salt marshes. The seeds were then mixed and stored at 4°C in a refrigerator. No specific permissions were required to perform these activities in any of the locations because these locations are not privately owned or protected in any way, and the field studies did not involve endangered or protected species.

### Greenhouse Experiment

The laboratory experiment was conducted in a greenhouse. In early April 2011, we collected cuttings of the invasive species. The cuttings were obtained from three different local communities in Guangzhou with no salinity stress and were similar in length (5 cm) and diameter (4 mm). Each cutting had two nodes and buds at each end, and cuttings were planted in nursery trays filled with moist sand. Concurrently, we selected seeds of similar size from each species, which were then sown in nursery trays and covered with moist sand. The sand was kept saturated by adding water every day.

After two weeks of growth, when the seedlings of each species were approximately 5 cm in height (for the invasive species, this refers to the shoots that emerged from the buds), each seedling was transplanted into a plastic pot (diameter 20 cm; height 15 cm) with a small hole in the bottom. The pots were filled with sand, soil and peat in a 1∶1:1 proportion by volume. Before the seedlings were transplanted, a woody supporting frame (2 m in height) was inserted into each pot for trailing the vines. Finally, the pots were placed in a shallow plastic tray. There were 400 pots in total, of which 40 (4 species × 10 replicates) were prepared for calculating the initial biomass prior to the stress treatments. The remaining 360 pots were prepared for the experimental treatments. All the pots were randomly arranged in the greenhouse, and their locations were changed once per week. Seedlings may be initially sensitive to the salinity treatment; thus, every pot was irrigated with 200 ml of water every day before the stress treatment began. Twenty-eight individuals (14 *I. cairica*, 1 *I. triloba*, 10 *I. digitata*, 3 *P. foetida*) died at the beginning of the experiment, which may have occurred because the seedlings were not large enough and the roots were damaged during transplantation.

Approximately one month after transplantation, when the seedlings were large enough, 10 pots of each species (40 pots) were randomly selected for calculating the initial biomass, and the leaves, stems and roots of the seedlings were harvested. The stem length and dry biomass of the leaves, stems and roots were measured for each plant. Concurrently, the salinity and simulated herbivory treatments began. The prepared plants of each species were randomly separated into 9 groups. The initial stem length of each individual was measured at the beginning of the treatments. The nine experimental units were set as three salinity stress gradients (0, 4 and 8 g L^−1 ^NaCl solution) × three simulated herbivory gradients (0, 25 and 50% of the leaf area excised) (see Figure S1 for details). The salinity treatments were applied by watering the plants with 200 ml of pure water (0 g L^−1 ^NaCl), 4 g L^−1^ or 8 g L^−1 ^NaCl solution. The treatments were applied every other day until the end of the experiment. The simulated herbivory treatments were conducted by excising 0, 25 or 50% of the total leaf area of each plant using scissors. We then immediately sprayed the plants that had been clipped with a 1 mmol L^−1^ solution of jasmonic acid (J2500, Sigma Chemical Co., St Louis, Missouri, USA) until they were dripping [57]. Jasmonic acid is a natural elicitor of defenses against herbivores, and a clipping treatment in combination with jasmonic acid application appears to be a good method for the induction of responses similar to those that are induced by natural herbivores [58]. A jasmonic acid solution of 1 mmol L^−1^ is thought to be useful for plant induced defense [59]. In our experiments, the jasmonic acid was first dissolved in methanol and subsequently diluted with distilled water to achieve the required concentration. The plants that were not clipped were sprayed with the solvent (methanol and distilled water) until dripping. This treatment was conducted twice during the experiment: once at the beginning of the treatments and once in mid-July.

In mid-August, all parts of the plants were harvested and leaf areas were measured immediately. The dry biomass was measured after the samples had been dried to a constant weight (for approximately 48 h) in an oven at 60°C.

### Measurements

To evaluate the level of inducible defense, we determined the condensed tannin content of leaves because condensed tannin from sugars is known to be elicited by both wounding and jasmonic acid [60]. Condensed tannin may function to deter grazers, reduce digestion efficiency, and inhibit the growth of microbes [61], [62], [63]. Before the final harvest, we obtained 10 g fresh leaves (fresh weight) from each plant for determining the condensed tannin content (CT%) of the plants under different treatments. The fresh leaf samples were cut, immediately placed on ice, and transported to the laboratory under dark conditions. The fresh samples were freeze-dried using a freeze-drying system (Free Zone 6L, LABCONCO, Kansas City, MO, USA) and subsequently ground at 4°C in a refrigerator until all of the samples passed through a 0.5 mm sieve. The HCl-butanol assay method was used for determining the condensed tannin content in the samples as described by FAO and IEAE [64], and the content was calculated as the percentage of condensed tannin in the dry leaves (CT%).

The stem length (x) and total biomass (y) of the plants that were harvested at the beginning of the treatments were used for fitting regression equations for each species (*I. cairica*, y = 0.088e^0^.^011x^, R^2^ = 0.960; *I. triloba*, y = 0.00005x^2^–0.019x +2.829, R^2^ = 0.897; *I. digitata*, y = 0.010x +0.324, R^2^ = 0.973; *P. foetida*, y = 0.005x–0.218, R^2^ = 0.936). Then, the initial biomass of each of the remaining individuals was calculated using these regression equations and the initial stem lengths.

The dry weights of the roots, leaves and stems were determined. The relative growth rates (RGR) were calculated using the initial estimates of biomass based on the plants harvested prior to the treatments and the final harvested biomass, following Hunt [65]:
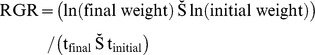



The tolerance score was calculated following Huang *et al.* [30], and the formula was developed to equal the ratio of the total biomass of stressed plants divided by the mean value of the untreated controls.
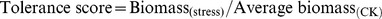



Biomass _(stress)_ indicates the biomass of an individual of a species that has undergone stress treatments (i.e., simulated herbivory, salinity stress or both). Average biomass _(CK)_ indicates the average biomass of the species under the control treatment.

In this experiment, the biomass that was removed from the plants that were subjected to simulated herbivory was not added to the final harvested biomass, in line with previous studies [66], [67]. We believe that considering the harvested biomass without the removed biomass is appropriate for this study because our aim is to examine whether plants can compensate for the biomass losses caused by simulated herbivory (i.e., tolerance).

### Statistical Analyses

Kolmogorov-Smirnov tests were first used to examine the normality of residuals for all analyses. The data were transformed using the natural logarithm to obtain a normal distribution. To evaluate whether plant growth was affected by the treatments, a multivariate analysis of variance (MANOVA) was conducted with salinity and simulated herbivory treatments as fixed factors and leaf biomass, stem biomass, root biomass and leaf area as dependent variables (see Tables S1 and S2 for further information).

The invasive and non-invasive plants were cultivated by cuttings and seeds, respectively. The results of the comparisons between their initial weights showed a significant difference (*F = *488.26, Num *df = *3, Den *df = *328, *P*<0.001). This difference in initial biomass may lead to biased results. Thus, the initial weight of the plants was used as a covariate for a 3-way ANCOVA (dependent variable: RGR; fixed factors: species, salinity stress and simulated herbivory) to detect which factor significantly influenced RGR. All the possible interactions among explanatory variables/fixed factors were tested. Bonferroni corrections based on marginal means were selected for post hoc tests to determine whether the RGR values of the invasive species were always highest across 9 treatments (CK, simulated herbivory 25% (SH_low_), 50% (SH_high_), salinity stress 4 g L^−1^ NaCl (SS_low_), 8 g L^−1^ NaCl (SS_high_), SS_low_ + SH_low_, SS_low_ + SH_high_, SS_high_ + SH_low_ and SS_high_ + SH_high_). A similar procedure for 3-way ANOVA was used for the analysis of plant chemical resistance. All data from the stress treatments were used for species tolerance comparison (one-way ANCOVA and Bonferroni multiple comparison; covariate: initial weight). The comparisons were separately executed under three scenarios (treatments with salinity, treatments with simulated herbivory and treatments with both of the two factors simultaneously) using the tolerance scores of all plants under each scenario.

In some analyses, the degrees of freedom differed among species, which was caused by unequal final numbers of plants per species. In the MANOVA analysis, the value of *Error df* is not an integer because these degrees of freedom are calculated using the mean squared errors. All analyses were conducted with SPSS 16 for Windows (SPSS Inc, Chicago, Illinois).

## Results

### Plant Biomass Growth Performance

**Figure 1:**
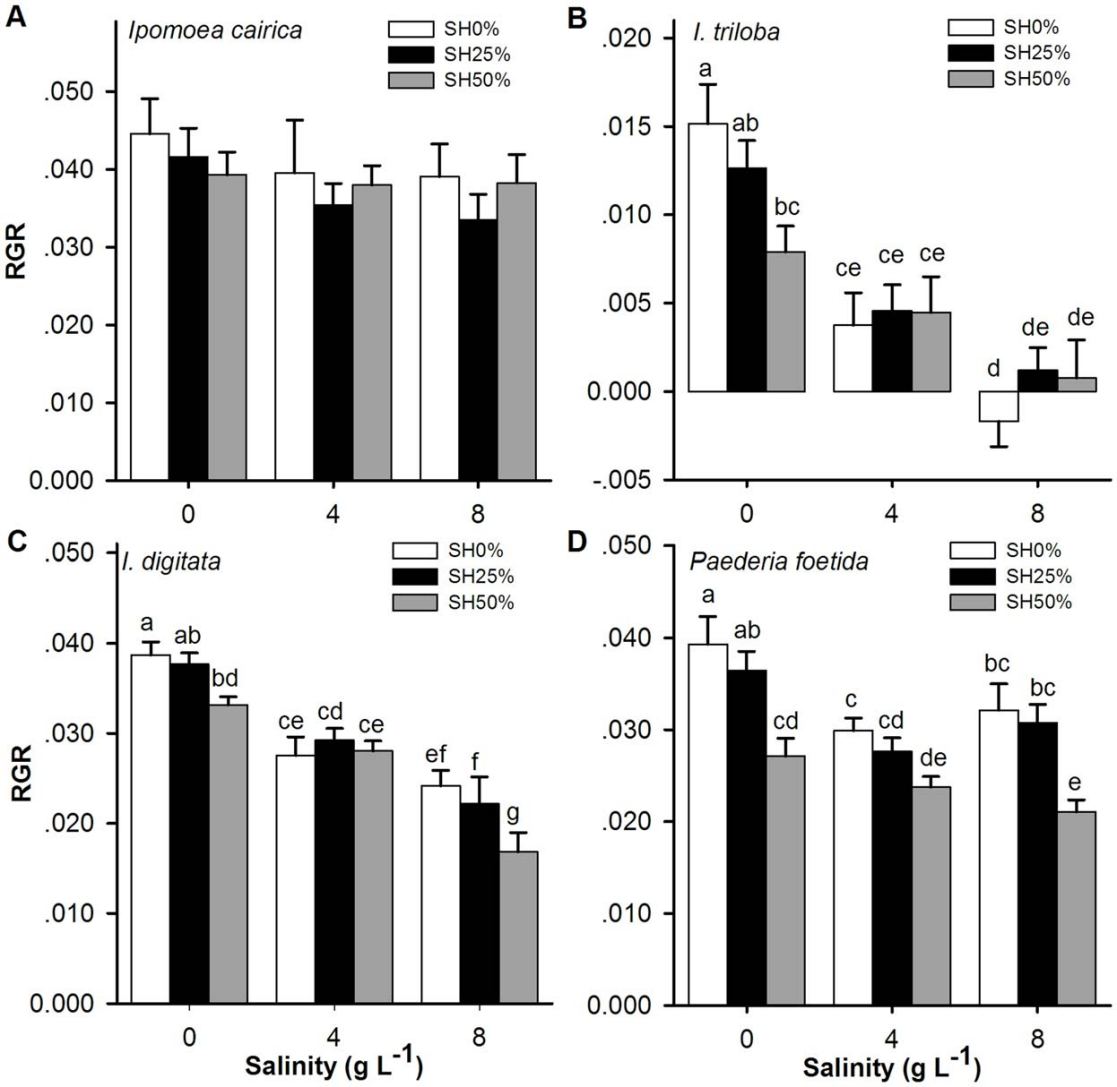
The relative growth rates (RGRs) of the 4 species influenced by salinity stress and simulated herbivory. Legends (SH0%, SH25% and SH50%): 0, 25 and 50% of the leave area of the plant was removed by simulated herbivory. Salinity stress: watered with 0, 4 and 8 g L^−1^ NaCl solution. Bonferroni corrections were used for the multiple comparison analyses, and significant differences (at the significance level of α = 0.05) are considered to be present between two treatments with different symbols. Error bars represented Mean ± SE (see Table S3 for supplementary test statistics).

The MANOVA analysis (Table 1) indicated that salinity stress, simulated herbivory and their interactions had highly significant effects on the growth of native and exotic non-invasive plants. Moreover, their RGRs generally decreased along the gradient of the stress factors (Bonferroni multiple comparison, Fig. 1). However, the growth performance of the invasive plant *I. cairica* was not affected by salinity stress treatments or simulated herbivory treatments either separately or when both of the treatments were applied simultaneously (Wilks’ λ Value = 0.76, *Effect df = *8 or 16, *P*>0.05). The invader *I. cairica* maintained a stable RGR across all treatments (Fig. 1). The result of the 3-way ANCOVA (Table 2) revealed that RGR was significantly influenced by species, the two stress factors and the two types of interactions (species × salinity and species × simulated herbivory). Further analysis (Bonferroni multiple comparisons, Table 3 and Fig. 1) revealed that the RGR of *I. cairica* was higher than that of the non-invasive species under high-stress treatments (i.e., salinity stress of 8 g L^−1^ NaCl, simulated herbivory of 50% and both treatments simultaneously). However, when there was no stress factor (CK), the RGR of the invader was only higher than that of the exotic non-invasive *I. triloba* and was not significantly different from that of the two native species. The RGRs of the exotic non-invasive *I. triloba* were lowest whether these plants were exposed to stress factors or not.

**Table 1:** Results of the MANOVA show the influence of stress factors on the growth performances of the four species.

Species	Stress factors	Wilks’ λ Value	*F*	*Effect df*	*Error df*	*P*
***Ipomoea cairica***	Salinity	0.80	1.84	8	128	0.075
	Simulated herbivory	0.85	1.36	8	128	0.219
	Salinity × Simulated herbivory	0.75	1.24	16	196.16	0.241
***I. triloba***	Salinity	0.15	30.46	8	154.00	0.000
	Simulated herbivory	0.72	3.40	8	154.00	0.001
	Salinity × Simulated herbivory	0.58	2.91	16	235.88	0.000
***I. digitata***	Salinity	0.20	21.48	8	136.00	0.000
	Simulated herbivory	0.52	6.56	8	136.00	0.000
	Salinity × Simulated herbivory	0.48	3.49	16	208.38	0.000
***Paederia foetida***	Salinity	0.66	4.29	8	150.00	0.000
	Simulated herbivory	0.59	5.73	8	150.00	0.000
	Salinity × Simulated herbivory	0.63	2.35	16	229.77	0.003

Leaf biomass, stem biomass, root biomass and leaf area were the dependent variables, salinity stress and simulated herbivory were the fixed factors. Wilks test was used for this analysis.

**Table 2:** The results of the 3-way ANCOVA on the relative growth rates (RGRs).

	SS	MS	*df*	*F*	*P*
**Intercept**	0.0359	0.0359	1	678.96	0.00
**Initial biomass**	0.0002	0.0002	1	4.14	0.04
**Species**	0.0066	0.0022	3	41.82	0.00
**Salinity**	0.0052	0.0026	2	49.05	0.00
**Simulated herbivory**	0.0010	0.0005	2	9.88	0.00
**Species × Salinity**	0.0011	0.0002	6	3.44	0.00
**Species × Simulated** **herbivory**	0.0009	0.0002	6	2.83	0.01
**Salinity × Simulated** **herbivory**	0.0003	0.0001	4	1.58	0.18
**Species × Salinity ×** **Simulated herbivory**	0.0003	0.00002	12	0.46	0.94
**Error**	0.0156	0.0001	295		

The initial biomass of the 4 species was used as covariate for 3-way ANCOVA.

**Table 3:** The significance for comparing the difference of the relative growth rates (RGR)/the condensed tannin content (CT%) among the 4 species under 9 different types of treatments.

Treatments		*I. cairica*	*I. triloba*	*I. digitata*	*P. foetida*
**CK**	RGR	a	b	a	a
	CT%	a	c	a	b
**Simulated herbivory (low)**	RGR	a	b	a	a
	CT%	a	b	a	b
**Simulated herbivory (high)**	RGR	a	c	ab	b
	CT%	a	b	a	b
**Salinity (low)**	RGR	a	b	a	a
	CT%	a	c	b	c
**Salinity (high)**	RGR	a	c	b	ab
	CT%	a	bc	b	c
**Salinity (low) + Simulated herbivory (low)**	RGR	a	c	ab	b
	CT%	a	b	a	b
**Salinity (low) + Simulated herbivory (high)**	RGR	a	c	b	b
	CT%	a	c	a	b
**Salinity (high) + Simulated herbivory (low)**	RGR	a	c	b	ab
	CT%	a	b	a	b
**Salinity (high) + Simulated herbivory (high)**	RGR	a	c	b	b
	CT%	a	b	a	a

Bonferroni corrections were used for the multiple comparison analyses, and significant differences (at the significance level of α = 0.05) are considered to be present between two species with different symbols. Treatments: CK, treatments with no salinity and no simulated herbivory stress; simulated herbivory (low) or simulated herbivory (high), treatments with 25% or 50% leaf area cutting; salinity (low) or salinity (high), treatments with watering 4 g L^−1^ or 8 g L^−1^ NaCl solution; “+”, treatments with both salinity and simulated herbivory stress simultaneously.

### Tolerance to Stress Factors

**Figure 2:**
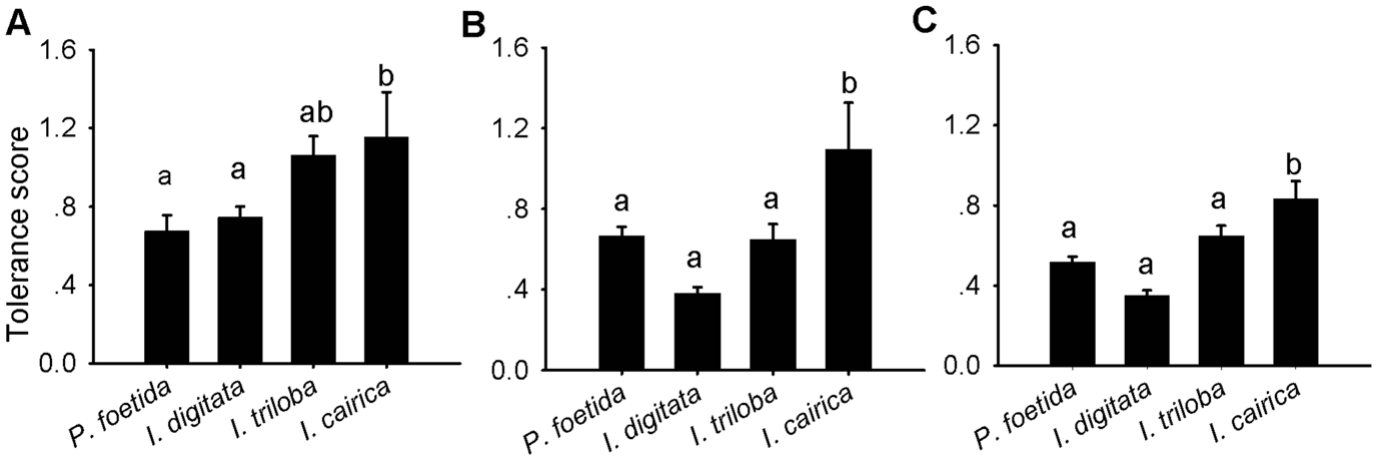
Comparing the tolerance scores of the 4 species under stress treatments. (A) Treatments with simulated herbivory; (B) treatments with salinity stress; (C) plants subjected to both salinity and simulated herbivory stress simultaneously. The Bonferroni correction was used for multiple comparison analyses; different characters indicate significant differences (significance level α = 0.05). Error bars represented Mean ± SE.

The results of the one-way ANCOVA revealed that the tolerance to stress factors was significantly different among the species tested (*F = *17.47, Num *df = *3, Den *df* = 327, *P*<0.001). Further comparisons revealed that the species tolerance scores were significantly different across three types of stress treatments: simulated herbivory stress only (*F = *4.40, Num *df = *3, Den *df* = 68, *P* = 0.007), salinity stress only (*F = *11.59, Num *df = *3, Den *df* = 66, *P*<0.001), and salinity stress and simulated herbivory simultaneously (*F = *18.12, Num *df = *3, Den *df* = 142, *P*<0.001). The invasive species *I. cairica* exhibited the highest tolerance to the two stress factors regardless of scenario (Fig. 2), and the tolerance scores of the three non-invasive species were not significantly different from each other. The exotic non-invasive *I. triloba* maintained a relatively high tolerance score when only simulated herbivory was added.

### Chemical Defense and Condensed Tannin Content

**Figure 3:**
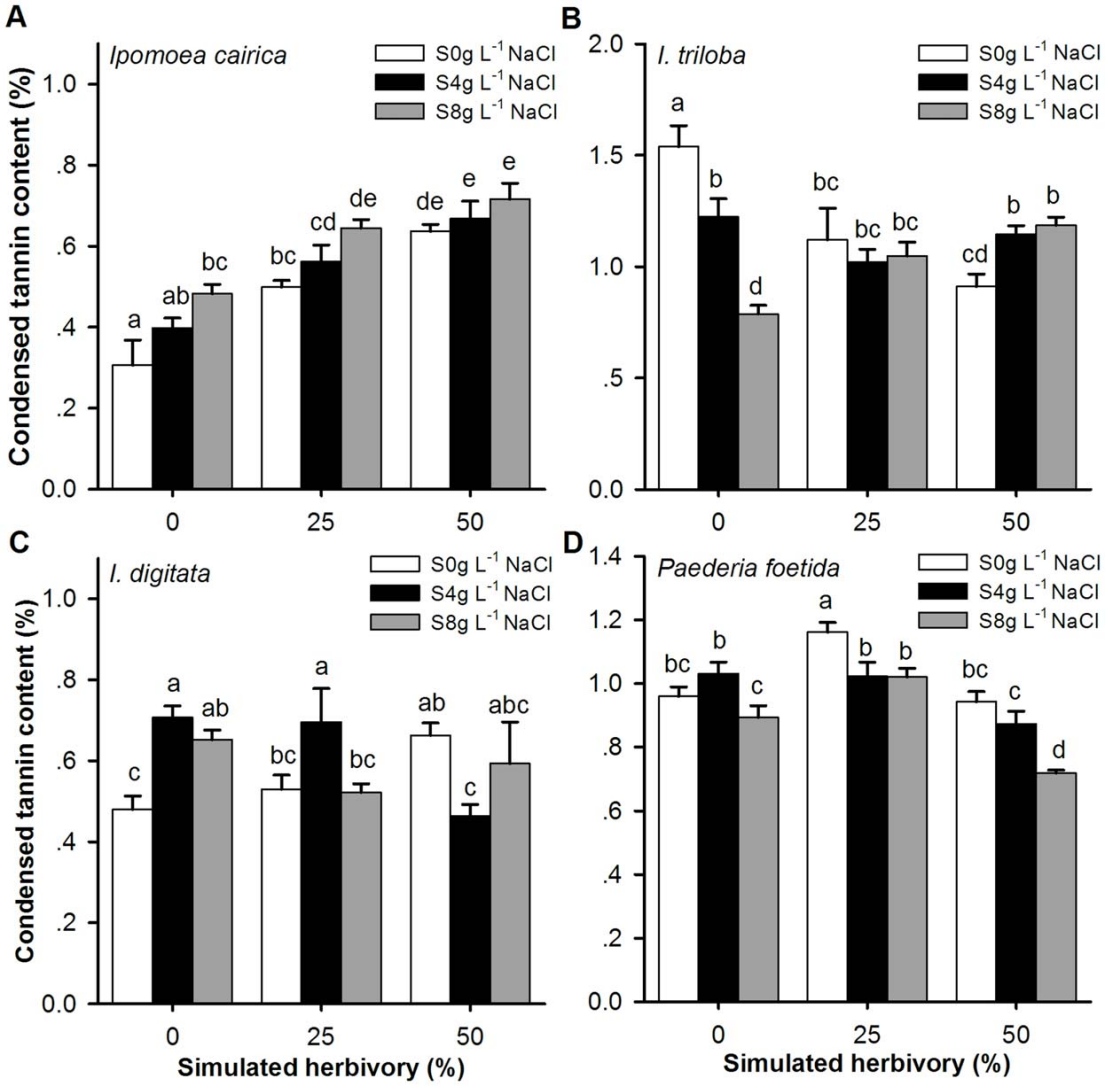
The condensed tannin content (CT%) of the species influenced by salinity stress and simulated herbivory. Legends (0 g L^−1^ NaCl, 4 g L^−1^ NaCl and 8 g L^−1^ NaCl): salinity stress, watered with 0, 4 and 8 g L^−1^ NaCl solutions; simulated herbivory treatments: 0, 25 and 50% of the leaf area excised. Bonferroni corrections were used for the multiple comparison analyses, and significant differences (at the significance level of α = 0.05) are considered to be present between two treatments with different symbols. Error bars represented Mean ± SE (see Table S4 for supplementary test statistics).

The results of the 3-way ANOVA (Table 4) revealed that the condensed tannin content (CT%) was significantly influenced by species, simulated herbivory and the interactions between these factors. Furthermore, the CT% of the invasive species was significantly lower than that of the non-invasive species, with the exception of *I. digitata*, under all scenarios (Fig. 3 and Table 3). However, under the most stressful treatment (i.e., salinity at 8 g L^−1 ^NaCl + simulated herbivory at 50%), the CT% of the invader was only significantly higher than that of *I. triloba*. However, the CT% of the exotic non-invasive *I. triloba* was the highest in almost all scenarios (Fig. 3 and Table 3).

**Table 4:** The result of 3-way ANOVA on the CT%.

	SS	MS	*df*	*F*	*P*
**Intercept**	105.86	105.86	1	8720.87	0.000
**Species**	10.43	3.48	3	286.34	0.000
**Salinity**	0.05	0.023	2	1.88	0.160
**Simulated herbivory**	0.08	0.04	2	3.29	0.043
**Species × Salinity**	0.61	0.10	6	8.39	0.000
**Species × Simulated herbivory**	0.69	0.12	6	9.45	0.000
**Salinity × Simulated herbivory**	0.25	0.06	4	5.21	0.001
**Species × Salinity × Simulated herbivory**	1.64	0.14	12	11.27	0.000
**Error**	0.87	0.01	72		

## Discussion

The exotic invasive species *I. cairica* is expanding to salt marshes in its introduced range although herbivore pressure from native insects simultaneously exists to some extent. According to our survey in South China, *I. cairica* has recently expanded to similar high salinity habitats in various places including Zhuhai, Shenzhen and Zhongshan. Although *I. cairica* has been invading non-saline habitats for approximately 100 years, this uncommon expansion to a high salinity stressed habitat has only been observed in recent years. Thus, the recent expansion to salt marshes is not a singular or locally restricted event. Our results reveal that the expansion of *I. cairica* to a more stressful habitat was facilitated by its relatively high tolerance to stress factors (primarily salinity stress and herbivory) in the new habitat.

Due to the energetic costs of dealing with environmental stress, plant fitness may be unfavorably impacted by various stress factors [38]. Plants in stressful environments commonly display low rates of photosynthesis and growth [68]. The growth of plants in habitats with more than one stress factor may be particularly negatively influenced due to interactions between the stress factors. For example, herbivory decreased seedling survival and growth of temperate rainforest species in the shade, but this did not happen in the sun [69]. Previous studies revealed that the salinity tolerance of invaders is important because it may help to relate physiological tolerance to distribution limits in the field [70]. High tolerance to herbivory is also believed to be an important characteristic of a successful invasion [29], [35]. The previously mentioned studies usually examined the tolerance of an invader for a single stress factor. However, for invaders in the secondary invasion phase, stressful environmental factors may be simultaneously encountered more frequently [22]. Tolerance may be an effective way to address various stressors simultaneously because similar compensatory growth mechanisms may be activated after the destruction of apical meristems (i.e., the tip of a growing stem or branch) by various stress and disturbance factors [71]. Therefore, effective tolerance mechanisms may be common in plant invaders [72], especially for species in the secondary phase of invasion. However, in the context of plant invasion, tolerance has received little attention [72]. In this study, the RGRs of the non-invasive species were decreased, whereas the RGR of the invasive species was not significantly influenced by the two stress factors. Thus, invasive species maintain a higher tolerance to environmental stress factors than native and exotic non-invasive congeners. Other studies also have found that invasive species have a higher tolerance to various stress factors compared with native species [73], [74], [75], [76], [77] and their native conspecifics [24], [78]. Due to their high tolerance to salinity [37] and herbivory stress [35], some invasive species have successfully invaded harsh habitats. Taken together, these results strongly support our hypothesis. The growth performance of *I. triloba* was significantly influenced by herbivory and salinity stress (Table 1). This finding suggests that this exotic non-invasive species was sensitive to the two factors. The relatively low stress tolerance of *I. triloba* may be one of the major reasons why it has not invaded to a high degree just like *I. cairica*. The native species *P. foetida*, which is a significant invader in North America, also maintains a low level of tolerance to these stress factors, although it may be more vigorous in its non-indigenous habitat.

The RGR of *I. cairica* was generally higher than that of the non-invasive species when high levels of stress factors were present (Fig. 1 and Table 3). These data suggest that *I. cairica* can maintain a fast growth rate even in stressful environments. These traits are considered to be important factors in a successful invasion [9], [79], [80].

The invasive species were cultivated from cuttings, whereas the non-invasive species were cultivated from seeds, and the difference in initial size might lead to a biased result. Therefore, we used the initial biomass as a covariate in the 3-way ANCOVA (Table 2). Although the result showed that the RGR was significantly influenced by initial biomass (*F = *4.14, *df = *1, *P = *0.04), significant influences from species and stress factors were also detected in the corrected analysis. Subsequent analysis (Fig. 1) revealed that the RGR of the invader was not significantly influenced by stress factors; however, the RGRs of the non-invasive species were significantly influenced. These findings suggest that the tolerance of the invader to stress factors was indeed higher than that of the non-invasive species when we corrected for the effect of initial size. Although the analysis was corrected using the initial plant biomass, we acknowledge that this may not eliminate the differential effects caused by seeds and cuttings because the environmental carry-over effects of plants cultivated from cuttings may be stronger than those of plants cultivated from seeds, which mainly rely on maternal effects. Indeed, if *I. cairica* plants could be raised from seeds, this would eliminate differential effects and enhance the validity of our results. However, because *I. cairica* mainly reproduces through vegetative propagation, seed material was not sufficient for the experiments. Additionally, the seeds of the non-invasive species were sampled from a wider geographic range and a larger number of populations than the vegetative *I. cairica* cuttings. Although this is an essential part of the experimental design that leads to drawbacks, we believe that our results are meaningful and important for understanding the secondary invasion of *I. cairica*.

Invasive species may use different strategies for resource allocation compared with native and exotic congeners. When encountering an environmental stressor, plants face a trade-off in resource allocation among growth maintenance, resistance [81], tolerance [82], [83] and other responses. The relatively low leaf condensed tannin content but significantly high growth rate (Fig. 1, Fig. 3 and Table 3) and tolerance scores (Fig. 2) indicate that *I. cairica* has allocated more energy to maintaining growth and tolerance but less for resistance compared with non-invasive species. For many plants, condensed tannins are often accumulated in response to herbivory [84], [85], [86]. Defense behavior is costly to plant growth and fitness [87]. When a plant is attacked by herbivores, it faces a trade-off between investment in the production of secondary compounds and investment in growth [88] and tolerance [82], [83]. Previous research has revealed that many plants face a trade-off when defoliated because they can either invest in rapid re-growth of relatively “cheap” photosynthetic tissue to compensate for the lost leaf biomass or in more “expensive” leaves that contain a higher concentration of secondary metabolites to deter herbivores [88], [89]. If invasive species decrease resource allocation to costly defenses as posited by the ERH, they would maintain enough resources to increase allocation to growth and competitive ability as described by the EICA hypothesis (Evolution of Increased Competitive Ability) [90]. Thus, the relaxation of herbivory pressure would quickly select for reduced expression of resistance traits in plants [91]. Some researchers have found that invasive species are less resistant to herbivory than conspecifics in their native ranges [24] but more tolerant to herbivore damage than their native relatives [29] and their conspecifics in their native ranges [24]. Although this shift has been detected, we still do not fully understand how altered patterns of selection might influence the evolution of plant tolerance during the invasion process [72]. Presumably, reduced herbivory pressure may contribute to growth and tolerance by allowing plants to use the energy that was previously allocated for defense to increase growth [92]. Additionally, allocating resources to tolerance may be more efficient than resistance for maintaining stable fitness when invaders simultaneously encounter various stress factors, especially during secondary invasion.

Our results may indicate that *I. cairica* in introduced habitats with reduced herbivory pressure would evolve reduced levels of costly resistance traits [93] and increased levels of tolerance traits during its relatively long invasion history. A study on the invasive tree *Sapium sebiferum* revealed that Chinese ecotypes (from the native range) were negatively affected by severing roots, but Texas ecotypes (from the introduced range) were able to compensate for root damage, indicating that the invader has undergone a shift away from costly herbivory defense and initiated the production of relatively inexpensive tissues that are capable of rapidly compensating for damage [94]. However, we do not yet understand whether the condensed tannin content in this invasive population would also be lower than that in its original range. Thus, we cannot arbitrarily conclude that there was a shift in trade-offs. However, we deduce that *I. cairica* may have evolved to reduce defense and increase tolerance as the result of natural selection, e.g., release from enemies in the primary invasion phase. Additional studies are needed to support this viewpoint directly by comparing genotypes from the native and introduced ranges.

In fact, although the condensed tannin content of *I. cairica* was lower than that of *I. triloba* and *P. foetida* under most of the treatments, an obvious induced chemical resistance [95] was observed due to a significantly increased condensed tannin content along the simulated herbivory gradients (Fig. 3). However, the condensed tannin content of the remaining three non-invasive species generally decreased along the herbivory gradient (Fig. 3) because the stress was too severe. Therefore, under the harshest treatment (salinity of 8 g L^−1^ NaCl + simulated herbivory of 50%), the condensed tannin content of the invader was not significantly lower than that of the two native species (Fig. 3 and Table 3). However, the RGR of the invader was still significantly higher than that of the two native species (Fig. 1 and Table 3). This finding may indicate that *I. cairica* can simultaneously maintain the highest RGR and a relatively high level of defense in a harsh environment.

Coastal salt marshes are rather productive ecosystems [96], and they are considered to be areas of high conservation and functional value [97]. However, coastal areas are endangered by numerous human impacts [98]. Salt marshes are characterized by high salinity stress and abundant resources, and the response of plants to salinity is determined by their general growth characteristics and physiological mechanisms of salt tolerance [36]. To date, we do not fully understand how the invasive species *I. cairica* copes with the high salinity stress in coastal salt marshes. Furthermore, evolutionary change is increasingly being recognized as an important factor contributing to the success of exotic plant invaders [94]. With respect to the long invasion history of *I. cairica*, environmental selection may favor genotypes that are more capable of enduring stressful environmental conditions, provided that there is sufficient genetic variation in the invasive populations [12]. However, we have not yet documented such evolutionary information on *I. cairica*. The processes driving plant invasions are likely to vary across different habitats [99] and over time, i.e., they are dependent on whether the plant is in its primary or secondary invasion phase [22]. Therefore, future studies should consider evolution, environmental heterogeneity, invasion history and the invader’s impact on the biodiversity of salt marshes.

In conclusion, we suggest that *I. cairica* may spread further and successfully invade the salt marsh ecosystem from its usual non-saline terrestrial environment. The high tolerance of *I. cairica* to simultaneous exposure to two key stresses, namely salinity and herbivory, indicates that this invasive species may also be capable of invading other types of stressful environments. The low resistance and high tolerance of *I. cairica* to herbivory indicates that this invasive species will not be controlled by introducing predators or any other measure based on the ERH. Thus, other measures should be taken to protect this sensitive habitat from invasion by additional non-native species.

## Supporting Information

Figure S1 **The experimental design.** The nine experimental units for each species were set as three salinity stress gradients (0 g L^−1^, 4 g L^−1^and 8 g L^−1 ^NaCl solution) × three simulated herbivory gradients (0%, 25% and 50% of leave area cut).
(DOC)

Table S1 The estimated parameters of 4 growth performance traits of each species under 9 types of treatments.
(DOC)

Table S2 The between effects covariance among 4 growth performance traits.
(DOC)

Table S3 One-way ANOVA for examining the influence of different treatments to the relative growth rates (RGRs) of plant species (supplementary test statistics for Figure 1).
(DOC)

Table S4 One-way ANOVA for examining the influence of different treatments to the condensed tannin content (CT%) of plant species (supplementary test statistics for Figure 3).
(DOC)
