# Supplementary material for: High Tolerance to Salinity and Herbivory Stresses May Explain the Expansion of Ipomoea Cairica to Salt Marshes
Source: PLoS One. 2012 Nov 15;7(11):e48829. doi: 10.1371/journal.pone.0048829 (PMC3499518; doi:10.1371/journal.pone.0048829)
Supplement: Figure S1 — The experimental design. The nine experimental units for each species were set as three salinity stress gradients (0 g L−1, 4 g L−1and 8 g L−1 NaCl solution) × three simulated herbivory gradients (0%, 25% and 50% of leave area cut). (DOC) [file pone.0048829.s001.doc]

**
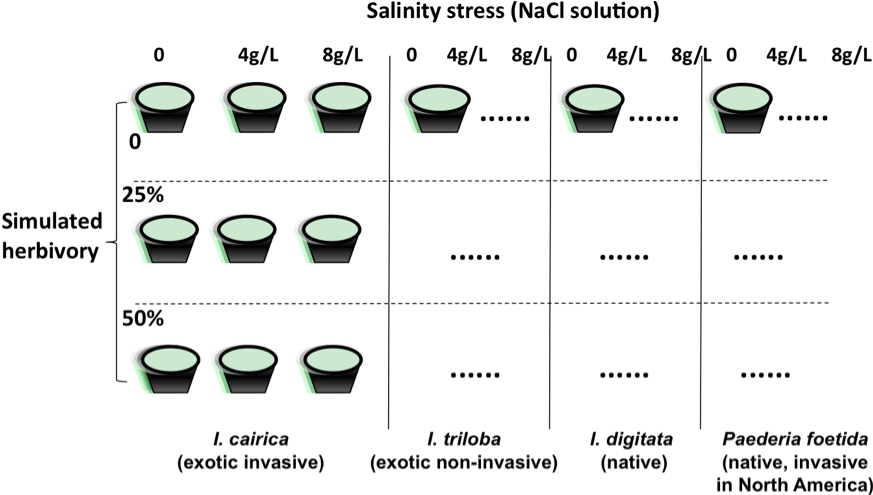
**

**Figure S1. The experimental design.** The nine experimental units for each species were set as three salinity stress gradients (0 g L^-1^, 4 g L^-1^and 8 g L^-1^ NaCl solution) × three simulated herbivory gradients (0%, 25% and 50% of leaf area cut).
