# Supplementary material for: High Tolerance to Salinity and Herbivory Stresses May Explain the Expansion of Ipomoea Cairica to Salt Marshes
Source: PLoS One. 2012 Nov 15;7(11):e48829. doi: 10.1371/journal.pone.0048829 (PMC3499518; doi:10.1371/journal.pone.0048829)
Supplement: Table S1 — The estimated parameters of 4 growth performance traits of each species under 9 types of treatments. (DOC) [file pone.0048829.s002.doc]

**Table S1.** The estimated parameters of 4 growth performance traits of each species under 9 types of treatments.

| **Species** | **Salinity** | **Simulated herbivory** | **Leaf biomass / g (Mean ± SE)** | **Root biomass / g (Mean ± SE)** | **Stem biomass / g (Mean ± SE)** | **Leaf area / cm^2^ (Mean ± SE)** | **N** |
| --- | --- | --- | --- | --- | --- | --- | --- |
| ***Ipomoea cairica*** | 0 | 0 | 0.91 ± 0.15 | 0.66 ± 0.11 | 1.66 ± 0.29 | 413.03 ± 62.50 | 10 |
|  | 0 | 25% | 0.55 ± 0.17 | 0.41 ± 0.13 | 1.32 ± 0.32 | 239.93 ± 69.87 | 8 |
|  | 0 | 50% | 0.47 ± 0.16 | 0.30 ± 0.12 | 1.02 ± 0.30 | 266.38 ± 65.88 | 9 |
|  | 4 g L^-1^ NaCl | 0 | 0.55 ± 0.18 | 0.33 ± 0.14 | 1.58 ± 0.35 | 313.26 ± 74.7 | 7 |
|  | 4 g L^-1^ NaCl | 25% | 0.35 ± 0.17 | 0.20 ± 0.13 | 0.81 ± 0.32 | 178.7 ± 69.87 | 8 |
|  | 4 g L^-1^ NaCl | 50% | 0.31 ± 0.16 | 0.27 ± 0.12 | 0.92 ± 0.30 | 177.13 ± 65.88 | 9 |
|  | 8 g L^-1^ NaCl | 0 | 0.40 ± 0.18 | 0.23 ± 0.14 | 1.16 ± 0.35 | 193.35 ± 74.7 | 7 |
|  | 8 g L^-1^ NaCl | 25% | 0.25 ± 0.16 | 0.16 ± 0.12 | 0.80 ± 0.30 | 139.35 ± 65.88 | 9 |
|  | 8 g L^-1^ NaCl | 50% | 0.32 ± 0.16 | 0.21 ± 0.12 | 1.20 ± 0.30 | 182.91 ± 65.88 | 9 |
| ***I. triloba*** | 0 | 0 | 0.99 ± 0.07 | 2.47 ± 0.27 | 0.91 ± 0.09 | 422.89 ± 29.85 | 10 |
|  | 0 | 25% | 0.78 ± 0.07 | 2.54 ± 0.27 | 1.01 ± 0.09 | 309.80 ± 29.85 | 10 |
|  | 0 | 50% | 0.71 ± 0.07 | 2.06 ± 0.27 | 0.61 ± 0.09 | 325.78 ± 29.85 | 10 |
|  | 4 g L^-1^ NaCl | 0 | 0.45 ± 0.07 | 1.96 ± 0.27 | 0.53 ± 0.09 | 269.23 ± 29.85 | 10 |
|  | 4 g L^-1^ NaCl | 25% | 0.31 ± 0.07 | 1.70 ± 0.29 | 0.46 ± 0.10 | 160.28 ± 31.46 | 9 |
|  | 4 g L^-1^ NaCl | 50% | 0.43 ± 0.07 | 2.00 ± 0.27 | 0.51 ± 0.09 | 260.15 ± 29.85 | 10 |
|  | 8 g L^-1^ NaCl | 0 | 0.11 ± 0.07 | 1.29 ± 0.27 | 0.37 ± 0.09 | 59.43 ± 29.85 | 10 |
|  | 8 g L^-1^ NaCl | 25% | 0.18 ± 0.07 | 1.39 ± 0.27 | 0.37 ± 0.09 | 106.36 ± 29.85 | 10 |
|  | 8 g L^-1^ NaCl | 50% | 0.23 ± 0.07 | 1.45 ± 0.27 | 0.42 ± 0.09 | 125.45 ± 29.85 | 10 |
| ***I. digitata*** | 0 | 0 | 1.54 ± 0.10 | 3.19 ± 0.29 | 4.33 ± 0.29 | 836.68 ± 45.96 | 10 |
|  | 0 | 25% | 0.98 ± 0.10 | 2.17 ± 0.31 | 5.08 ± 0.30 | 447.23 ± 48.20 | 10 |
|  | 0 | 50% | 0.86 ± 0.11 | 2.10 ± 0.32 | 2.87 ± 0.32 | 413.31 ± 50.81 | 9 |
|  | 4 g L^-1^ NaCl | 0 | 0.74 ± 0.11 | 2.22 ± 0.32 | 1.10 ± 0.32 | 402.14 ± 50.81 | 9 |
|  | 4 g L^-1^ NaCl | 25% | 0.71 ± 0.12 | 2.81 ± 0.34 | 0.91 ± 0.34 | 348.99 ± 53.89 | 8 |
|  | 4 g L^-1^ NaCl | 50% | 0.77 ± 0.11 | 2.15 ± 0.32 | 1.13 ± 0.32 | 363.58 ± 50.81 | 9 |
|  | 8 g L^-1^ NaCl | 0 | 0.52 ± 0.12 | 1.90 ± 0.34 | 0.71 ± 0.34 | 241.45 ± 53.89 | 8 |
|  | 8 g L^-1^ NaCl | 25% | 0.36 ± 0.12 | 1.83 ± 0.34 | 0.68 ± 0.34 | 169.23 ± 53.89 | 8 |
|  | 8 g L^-1^ NaCl | 50% | 0.26 ± 0.12 | 1.24 ± 0.34 | 0.38 ± 0.34 | 139.89 ± 53.89 | 8 |
| ***Paederia foetida*** | 0 | 0 | 3.16 ± 0.27 | 4.11 ± 0.32 | 1.04 ± 0.08 | 958.18 ± 68.93 | 10 |
|  | 0 | 25% | 2.13 ± 0.32 | 2.99 ± 0.38 | 0.66 ± 0.10 | 494.60 ± 82.39 | 7 |
|  | 0 | 50% | 2.09 ± 0.27 | 2.36 ± 0.32 | 0.61 ± 0.08 | 576.31 ± 68.93 | 10 |
|  | 4 g L^-1^ NaCl | 0 | 2.17 ± 0.27 | 2.74 ± 0.32 | 0.75 ± 0.08 | 621.30 ± 68.93 | 10 |
|  | 4 g L^-1^ NaCl | 25% | 1.55 ± 0.27 | 2.05 ± 0.32 | 0.64 ± 0.08 | 395.68 ± 68.93 | 10 |
|  | 4 g L^-1^ NaCl | 50% | 1.46 ± 0.27 | 2.04 ± 0.32 | 0.45 ± 0.08 | 422.89 ± 68.93 | 10 |
|  | 8 g L^-1^ NaCl | 0 | 1.81 ± 0.27 | 2.58 ± 0.32 | 0.53 ± 0.08 | 455.61 ± 68.93 | 10 |
|  | 8 g L^-1^ NaCl | 25% | 1.79 ± 0.27 | 2.52 ± 0.32 | 0.49 ± 0.08 | 487.43 ± 68.93 | 10 |
|  | 8 g L^-1^ NaCl | 50% | 1.15 ± 0.27 | 1.96 ± 0.32 | 0.30 ± 0.08 | 359.55 ± 68.93 | 10 |
