# Supplementary material for: High Tolerance to Salinity and Herbivory Stresses May Explain the Expansion of Ipomoea Cairica to Salt Marshes
Source: PLoS One. 2012 Nov 15;7(11):e48829. doi: 10.1371/journal.pone.0048829 (PMC3499518; doi:10.1371/journal.pone.0048829)
Supplement: Table S2 — The between effects covariance among 4 growth performance traits. (DOC) [file pone.0048829.s003.doc]

**Table S2.** The between effects covariance among 4 growth performance traits.

| **Species** | **Factors** | **Variable** | **Leaf biomass / g** | **Root biomass / g** | **Stem biomass / g** | **Leaf area**  **/ cm^2^** |
| --- | --- | --- | --- | --- | --- | --- |
| ***Ipomoea cairica*** | Salinity | Leaf biomass / g | 0.02 |  |  |  |
|  |  | Root biomass / g | 0.02 | 0.01 |  |  |
|  |  | Stem biomass / g | 0.02 | 0.01 | 0.02 |  |
|  |  | Leaf area / cm^2^ | 7.78 | 6.17 | 6.85 | 3176.91 |
|  | Simulated berbivory | Leaf biomass / g | 0.01 |  |  |  |
|  |  | Root biomass / g | 0.01 | 0.00 |  |  |
|  |  | Stem biomass / g | 0.02 | 0.01 | 0.04 |  |
|  |  | Leaf area / cm^2^ | 5.67 | 3.48 | 10.83 | 2625.87 |
|  | Salinity × Simulated berbivory | Leaf biomass / g | 0.00 |  |  |  |
|  |  | Root biomass / g | 0.00 | 0.00 |  |  |
|  |  | Stem biomass / g | 0.01 | 0.00 | 0.02 |  |
|  |  | Leaf area / cm^2^ | 1.36 | 1.03 | 2.46 | 717.43 |
| ***I. triloba*** | Salinity | Leaf biomass / g | 0.08 |  |  |  |
|  |  | Root biomass / g | 0.11 | 0.16 |  |  |
|  |  | Stem biomass / g | 0.05 | 0.08 | 0.04 |  |
|  |  | Leaf area / cm^2^ | 28.52 | 42.84 | 19.85 | 11154.18 |
|  | Simulated berbivory | Leaf biomass / g | 0.00 |  |  |  |
|  |  | Root biomass / g | 0.00 | 0.00 |  |  |
|  |  | Stem biomass / g | 0.00 | 0.00 | 0.00 |  |
|  |  | Leaf area / cm^2^ | 0.87 | 0.14 | -0.42 | 622.78 |
|  | Salinity × Simulated berbivory | Leaf biomass / g | 0.01 |  |  |  |
|  |  | Root biomass / g | 0.01 | 0.02 |  |  |
|  |  | Stem biomass / g | 0.00 | 0.01 | 0.01 |  |
|  |  | Leaf area / cm^2^ | 2.48 | 3.51 | 1.48 | 1291.95 |
| ***I. digitata*** | Salinity | Leaf biomass / g | 0.09 |  |  |  |
|  |  | Root biomass / g | 0.10 | 0.13 |  |  |
|  |  | Stem biomass / g | 0.46 | 0.40 | 2.56 |  |
|  |  | Leaf area / cm^2^ | 48.18 | 52.04 | 233.37 | 24706.91 |
|  | Simulated berbivory | Leaf biomass / g | 0.02 |  |  |  |
|  |  | Root biomass / g | 0.03 | 0.07 |  |  |
|  |  | Stem biomass / g | 0.02 | 0.07 | 0.11 |  |
|  |  | Leaf area / cm^2^ | 11.51 | 17.01 | 10.47 | 7385.58 |
|  | Salinity × Simulated berbivory | Leaf biomass / g | 0.02 |  |  |  |
|  |  | Root biomass / g | 0.03 | 0.08 |  |  |
|  |  | Stem biomass / g | 0.02 | -0.03 | 0.19 |  |
|  |  |  |
|  |  | Leaf area / cm^2^ | 10.23 | 19.26 | 6.90 | 6465.22 |
| ***Paederia foetida*** | Salinity | Leaf biomass / g | 0.14 |  |  |  |
|  |  | Root biomass / g | 0.14 | 0.15 |  |  |
|  |  | Stem biomass / g | 0.05 | 0.04 | 0.02 |  |
|  |  | Leaf area / cm^2^ | 38.76 | 38.31 | 12.54 | 10575.89 |
|  | Simulated berbivory | Leaf biomass / g | 0.12 |  |  |  |
|  |  | Root biomass / g | 0.15 | 0.18 |  |  |
|  |  | Stem biomass / g | 0.05 | 0.06 | 0.02 |  |
|  |  | Leaf area / cm^2^ | 35.26 | 42.72 | 13.10 | 11268.97 |
|  | Salinity × Simulated berbivory | Leaf biomass / g | 0.03 |  |  |  |
|  |  | Root biomass / g | 0.03 | 0.06 |  |  |
|  |  | Stem biomass / g | 0.01 | 0.01 | 0.00 |  |
|  |  | Leaf area / cm^2^ | 13.97 | 16.93 | 4.32 | 6786.68 |
