# Supplementary material for: High Tolerance to Salinity and Herbivory Stresses May Explain the Expansion of Ipomoea Cairica to Salt Marshes
Source: PLoS One. 2012 Nov 15;7(11):e48829. doi: 10.1371/journal.pone.0048829 (PMC3499518; doi:10.1371/journal.pone.0048829)
Supplement: Table S3 — One-way ANOVA for examining the influence of different treatments to the relative growth rates (RGRs) of plant species (supplementary test statistics for Figure 1). (DOC) [file pone.0048829.s004.doc]

**Table S3.** One-way ANOVA for examining the influence of different treatments to the relative growth rates (RGRs) of plant species (supplementary test statistics for Figure 1).

|  | **SS** | **MS** | ***df*** | ***F*** | ***P*** |
| --- | --- | --- | --- | --- | --- |
| ***P. foetida*** |  |  |  |  |  |
| **Intercept** | 0.076 | 0.076 | 1 | 1953.58 | 0.000 |
| **treatments** | 0.003 | 0.000 | 8 | 8.07 | 0.000 |
| **Error** | 0.003 | 0.000 | 78 |  |  |
| ***I. digitata*** |  |  |  |  |  |
| **Intercept** | 0.065 | 0.065 | 1 | 2507.10 | 0.000 |
| **treatments** | 0.004 | 0.000 | 8 | 17.96 | 0.000 |
| **Error** | 0.002 | 0.000 | 71 |  |  |
| ***I. triloba*** |  |  |  |  |  |
| **Intercept** | 0.003 | 0.003 | 1 | 85.12 | 0.000 |
| **treatments** | 0.002 | 0.000 | 8 | 10.12 | 0.000 |
| **Error** | 0.002 | 0.000 | 80 |  |  |
| ***I. cairica*** |  |  |  |  |  |
| **Intercept** | 0.113 | 0.113 | 1 | 889.20 | 0.000 |
| **treatments** | 0.001 | 0.000 | 8 | 0.75 | 0.648 |
| **Error** | 0.008 | 0.000 | 67 |  |  |
