# Supplementary material for: High Tolerance to Salinity and Herbivory Stresses May Explain the Expansion of Ipomoea Cairica to Salt Marshes
Source: PLoS One. 2012 Nov 15;7(11):e48829. doi: 10.1371/journal.pone.0048829 (PMC3499518; doi:10.1371/journal.pone.0048829)
Supplement: Table S4 — One-way ANOVA for examining the influence of different treatments to the condensed tannin content (CT%) of plant species (supplementary test statistics for Figure 3). (DOC) [file pone.0048829.s005.doc]

**Table S4. One-way ANOVA for examining the influence of different treatments to the condensed tannin content (CT%) of plant species (supplementary test statistics for Figure 3).**

|  | **SS** | **MS** | ***df*** | ***F*** | ***P*** |
| --- | --- | --- | --- | --- | --- |
| ***P. foetida*** |  |  |  |  |  |
| **Intercept** | 38.74 | 38.74 | 1 | 7552.21 | 0.000 |
| **treatments** | 0.58 | 0.07 | 8 | 14.14 | 0.000 |
| **Error** | 0.09 | 0.01 | 18 |  |  |
| ***I. digitata*** |  |  |  |  |  |
| **Intercept** | 14.66 | 14.66 | 1 | 1203.58 | 0.000 |
| **treatments** | 0.33 | 0.04 | 8 | 3.37 | 0.015 |
| **Error** | 0.22 | 0.01 | 18 |  |  |
| ***I. triloba*** |  |  |  |  |  |
| **Intercept** | 51.83 | 51.83 | 1 | 1964.07 | 0.000 |
| **treatments** | 1.68 | 0.21 | 8 | 7.97 | 0.000 |
| **Error** | 0.48 | 0.03 | 18 |  |  |
| ***I. cairica*** |  |  |  |  |  |
| **Intercept** | 11.06 | 11.06 | 1 | 2277.49 | 0.000 |
| **treatments** | 0.73 | 0.09 | 8 | 18.78 | 0.000 |
| **Error** | 0.09 | 0.00 | 18 |  |  |
